# A simple method for plasma total vitamin C analysis suitable for routine clinical laboratory use

**DOI:** 10.1186/s12937-016-0158-9

**Published:** 2016-04-21

**Authors:** Line Robitaille, L. John Hoffer

**Affiliations:** Lady Davis Institute for Medical Research and Department of Medicine, McGill University and Jewish General Hospital, 3755 Cote Sainte Catherine, Montreal, QC H3T 1E2 Canada

**Keywords:** Ascorbic acid, Ascorbic acid deficiency, Avitaminosis, Dehydroascorbic acid, Malnutrition, Vitamin C

## Abstract

**Background:**

In-hospital hypovitaminosis C is highly prevalent but almost completely unrecognized. Medical awareness of this potentially important disorder is hindered by the inability of most hospital laboratories to determine plasma vitamin C concentrations. The availability of a simple, reliable method for analyzing plasma vitamin C could increase opportunities for routine plasma vitamin C analysis in clinical medicine.

**Methods:**

Plasma vitamin C can be analyzed by high performance liquid chromatography (HPLC) with electrochemical (EC) or ultraviolet (UV) light detection. We modified existing UV-HPLC methods for plasma total vitamin C analysis (the sum of ascorbic and dehydroascorbic acid) to develop a simple, constant-low-pH sample reduction procedure followed by isocratic reverse-phase HPLC separation using a purely aqueous low-pH non-buffered mobile phase. Although EC-HPLC is widely recommended over UV-HPLC for plasma total vitamin C analysis, the two methods have never been directly compared. We formally compared the simplified UV-HPLC method with EC-HPLC in 80 consecutive clinical samples.

**Results:**

The simplified UV-HPLC method was less expensive, easier to set up, required fewer reagents and no pH adjustments, and demonstrated greater sample stability than many existing methods for plasma vitamin C analysis. When compared with the gold-standard EC-HPLC method in 80 consecutive clinical samples exhibiting a wide range of plasma vitamin C concentrations, it performed equivalently.

**Conclusion:**

The easy set up, simplicity and sensitivity of the plasma vitamin C analysis method described here could make it practical in a normally equipped hospital laboratory. Unlike any prior UV-HPLC method for plasma total vitamin C analysis, it was rigorously compared with the gold-standard EC-HPLC method and performed equivalently. Adoption of this method could increase the availability of plasma vitamin C analysis in clinical medicine.

## Background

The disease of terminal vitamin C deficiency – scurvy – is first suspected on clinical grounds. The diagnosis is confirmed by documenting a plasma vitamin C concentration < 11.4 μmol/L and observing prompt clinical improvement after appropriate vitamin C provision [1, 2]. Scurvy is rare in the modern world, but hypovitaminosis C (plasma vitamin C concentration < 28.4 μmol/L [3]) or marginal vitamin C deficiency (plasma vitamin C concentration < 28.4 μmol/L but > 11.4 μmol/L [3]) is not. Hypovitaminosis C occurs in ~ 10 % of the general population [4], in ~ 30 % of cigarette smokers [5, 6] and ~ 60 % of acutely hospitalized patients [7–14], in whom it could contribute to fatigue and mood disturbance [13–15], immune system dysfunction [7, 16, 17], impaired wound healing [18–20], the complex regional pain syndrome [21] and the complications of cardiovascular disease [22–26].

Vitamin C distributes rapidly throughout the body’s extracellular fluids. Systemic inflammation and intravenous fluid therapy increase the body’s extracellular fluid volume, with resultant lowering of the concentration of vitamin C in the bloodstream [27–29]. In-hospital hypovitaminosis C is not solely a phenomenon of extravascular redistribution, however [12, 29, 30]. Systemic inflammation ignites an oxidative process that accelerates cellular vitamin C uptake and catabolism and increases its nutritional requirement [31–33]. The best-studied human example of this phenomenon is cigarette smoking [20, 34, 35], which accelerates vitamin C catabolism [35], lowers its plasma and tissue concentrations [36, 37] and increases its nutritional requirement [6, 34]. The depleted plasma vitamin C concentration of cigarette smokers can be re-normalized either by smoking cessation [38] or vitamin C supplementation [34], both of which decrease circulating concentrations of the biomarkers of oxidative stress [39, 40]. Acutely hospitalized patients experience inflammatory-oxidative stresses that are much greater than the stress caused by cigarette smoke. A combined history of inadequate vitamin C intake and systemic inflammation strongly predicts hypovitaminosis C [10, 11, 41, 42].

The high prevalence, potentially serious adverse consequences and easy preventability of in-hospital hypovitaminosis C would normally motivate vigorous investigation of its clinical implications, especially since small randomized clinical trials of vitamin C supplementation in vitamin C-deficient patients suggest clinical benefit [7, 14, 43]. However, much greater physician awareness and many more clinical trials would be required to change the current bias of medical practice, which is to avoid prescribing vitamin C supplements even to patients whose dietary intake is inadequate and who are likely to be vitamin C-deficient.

It is a barrier to medical awareness of in-hospital hypovitaminosis C that very few clinical laboratories are equipped to detect it. Shipment of samples to reference laboratories is problematic because vitamin C is notoriously unstable, requiring rapid plasma stabilization and continuous storage at -80^o^ C [44, 45]. We attempted to surmount this barrier by developing a simple, accurate, robust and inexpensive plasma vitamin C method that could easily be set up and taken down in any normally equipped clinical laboratory.

**Fig. 1:**
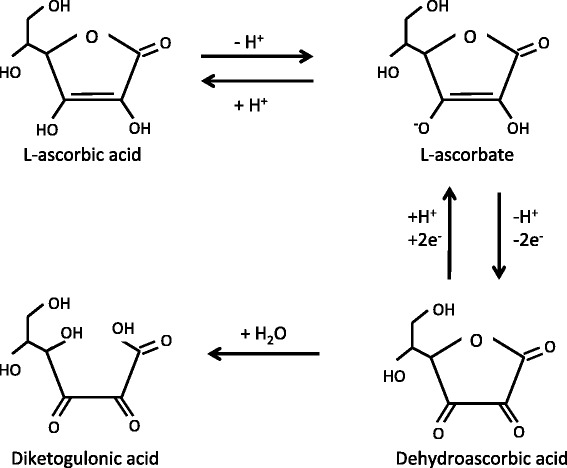
Vitamin C metabolism. Ascorbic acid participates both in acid-base (ascorbic acid-ascorbate, pK 4.2) and oxidoreduction reactions (ascorbate-dehydroascorbic acid). The latter reaction involves transient creation of the ascorbyl radical (not shown). Vitamin C (or total vitamin C) refers to the sum of ascorbic acid, ascorbate and dehydroascorbic acid. Under the strongly reducing, neutral pH conditions in the bloodstream, almost all vitamin C circulates as ascorbate. As soon as the blood cells are removed from plasma, ascorbate progressively oxidizes to dehydroascorbic acid, which is highly prone to irreversible hydrolysis to 2,3-diketogulonic acid. Diketogulonic acid is oxidized to smaller molecules, including oxalate, which are excreted in the urine. In order to minimize loss of vitamin C, freshly obtained plasma must immediately be acid-deproteinized and flash frozen, and strongly acidic conditions maintained as much as possible throughout sample storage and processing

The most sensitive and selective method for plasma vitamin C analysis uses high-pressure liquid chromatography (HPLC) coupled to an electrochemical (EC) detector [46–57]. EC-HPLC is invaluable in the research laboratory, but it requires costly, dedicated, high-maintenance equipment operated by specifically trained technicians. HPLC methods that require only an ordinary UV light detector are available [49, 51, 52, 56, 58–68] but their practicality in a non-specialized clinical laboratory is limited by a variety of characteristics: complicated mobile phase compositions with appreciable preparation and column conditioning times, dedicated columns, and complicated procedures to reduce dehydroascorbic acid to ascorbic acid in order to analyze total vitamin C, or inability to measure total vitamin C at all. Vitamin C consists of ascorbic acid and its redox partner, dehydroascorbic acid. The generic terms are used here, acknowledging that at physiological pH ascorbic acid is almost completely ionized and hence more correctly termed ascorbate (see Fig. 1). We incorporated recent improvements in food vitamin C analysis [69, 70] to develop a streamlined UV-HPLC plasma vitamin C analysis that is simple, sensitive and accurate enough for use in any normally equipped clinical laboratory.

EC-HPLC is commonly recommended as the method of choice for plasma vitamin C analysis [46, 49, 51, 56, 57], but no direct comparison of EC-HPLC and UV-HPLC methods for plasma total vitamin C have been reported. We therefore formally compared the simplified UV-HPLC method with EC-HPLC in 80 consecutive clinical samples.

## Methods

### Sample acquisition, handling, and storage

Venous blood (4 mL) was drawn into a 4 mL K_2_EDTA Vacutainer tube, mixed and immediately pushed into crushed ice and delivered to the laboratory for processing within one hour, as previously described [13, 14]. The plasma was separated in a refrigerated centrifuge (4^o^ C, 8 min, 2740 g) after which 0.2–0.4 mL of the resulting supernatant was immediately added to an equal volume of 10 % (w/v) metaphosphoric acid (MPA) in 2 mmol/L disodium EDTA, left on ice for 5 min, and cold-centrifuged (4^o^ C, 10 min, 16,000 g) as described by Lykkesfeldt [55]. The resulting protein-free acid supernatant was immediately flash-frozen in dry ice/ethanol and stored continuously at -80^o^ C until analysis either by EC- or UV-HPLC.

### EC-HPLC

The EC-HPLC method used to analyze ascorbic acid was closely similar to that of Levine, Wang and Rumsey [53]. Total vitamin C was analyzed using the sample reduction procedure described by Lykkesfedlt [54, 55]. We have used these reliable methods in our research laboratory for several years [13, 14, 71].

The analytical system consisted of an Agilent 1100 series HPLC system equipped with a Coulochem III electrochemical detector (ESA Inc) and a 5011A analytical cell. Electrode 1 was set to -175 mV, detector range 500 nA, and electrode 2 was set at 550 mV, detection range 50 μA. The HPLC column was a reverse phase Phenomenex Luna (4.6 x 250 mm, 5 μm) preceded by a SecurityGuard C18 cartridge. Column temperature was maintained at 25^o^ C. The mobile phase consisted of 25 % methanol and 75 % water containing 0.05 mol/L monobasic sodium phosphate, 0.05 mol/L sodium acetate trihydrate, 189 μmol/L dodecyltrimethylammonium chloride and 36.6 μmol/L tetraoctylammonium bromide, adjusted to pH 4.8 using orthophosphoric acid. The flow rate was 0.8 ml/min (isocratic). Retention times varied between 6 and 8 min depending on the extent of column conditioning. At the time of analysis, acidic protein-free plasma samples were thawed on ice in dim light and processed in two aliquots. To measure ascorbic acid, 50 μL of sample was mixed with 200 μL of water. The analysis of total vitamin C (ascorbic acid plus dehydroascorbic acid) required a reduction reaction in which 50 μL of sample was mixed with 25 μL of 2.5 mmol/L tris(2-carboxy ethyl) phosphine hydrochloride (TCEP) in 800 mmol/L TRIS buffer (pH 9) and allowed to stand in the dark for 5 min, after which 175 μL McIlvaine buffer (0.28 mol/L citric acid in 0.56 mol/L dibasic sodium phosphate, pH 4.5) was added. Both aliquots were centrifuged (4^o^ C, 5 min, 16,000 g) and the supernatants kept on ice and manually injected (20 μL) into the HPLC. At the end of each day’s sample run, the system was flushed with 25 % methanol in water in order to elute the buffers, but not so extensively as to affect ion-pairing conditions and alter analyte retention times.

The HPLC column required regeneration at 3–4 month intervals. The EC device was passivated with strong acid at 6–12 month intervals. These procedures required re-equilibration of the column and system, a process lasting a day or longer.

### UV-HPLC

The analysis was carried out using a Waters 2695 Separations Module equipped with a Waters 2487 dual wavelength UV detector set to 245 nm. The column was a reverse phase Agilent Zorbax Eclipse XDB-C18 (4.6 x 150 mm, 3.5 μm) fitted with an Agilent Eclipse XDB-C18 guard column. The column temperature was maintained at 25^o^ C. The mobile phase consisted of 1.8 mmol/L sulfuric acid (pH 2.7). The flow rate was 0.8 mL/min. The retention time was 3 min. There was a 7 min delay between injections to allow the uric acid peak to elute (5–6 min).

**Fig. 2:**
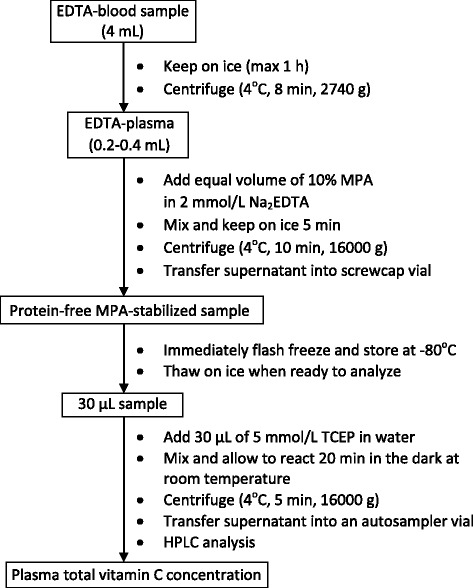
Sample preparation for plasma total vitamin C analysis by UV-HPLC. The individual steps are described in detail in the text

Zero-degree thawed samples were divided into two aliquots of 30 μL in 0.6 mL plastic Eppendorf tubes. To measure total vitamin C, an equal volume of 5 mmol/L TCEP in water (pH 2) was added and the sample allowed to react for 20 min at room temperature in the dark. To measure ascorbic acid, an equal volume of water was added instead of TCEP and the sample kept on ice. Both samples were then centrifuged (4^o^ C, 5 min, 16,000 g) and the supernatants kept on ice and transferred to autoinjection vials and either injected immediately onto the HPLC column (injection volume 20 μL) or kept in the refrigerated auto-sampler for up to 4 h. At the end of the day’s sample run, the system was rinsed with 40 % acetonitrile in water to remove the acidic mobile phase and any potential sample contaminants. Figure 2 summarizes the sample procedure for total vitamin C analysis.

For both methods, a standard curve was developed by using a peak areas linear regression equation from six ascorbic acid standards made up in MPA/EDTA ranging in concentration from 0 to 100 μmol/L. Each sample run included a plasma quality control sample.

### Patients

The UV-HPLC and EC-HPLC methods were used to analyze ascorbic acid and total vitamin C in plasma samples obtained from 80 consecutive clinical samples from patients attending an out-patient oncology clinic and enrolled in a study to evaluate their nutritional status. The protocol for that study was approved by the Research Ethics Committee of the Jewish General Hospital (clinicaltrials.gov registration #NCT01631526); its results will be reported separately.

### Statistical analysis

Statistical analyses were performed using Graph Pad Prism Version 5.01.

## Results

### Validation of the UV-HPLC method

A range of TCEP concentrations and reaction times was tested to verify the effectiveness of the sample reduction procedure described above, namely, 20 min incubation using 5 mmol/L TCEP at pH 2. Extension of the TCEP incubation period to 40 min left the results unchanged. The UV absorbance signal was linear both within the usual physiological range (5–100 μmol/L) and higher (r^2^ > 0.99). Sample spiking experiments within the physiological range (addition of 25 and 50 μmol/L known ascorbic acid to plasma samples with a concentration of 37 μmol/L) yielded total vitamin C recoveries of 106 % and 100 % respectively. The identity and specificity of the ascorbic acid chromatographic peak was verified by treating plasma samples with *ascorbate oxidase* and documenting complete disappearance of the ascorbic acid peak without any other change in the chromatogram.

**Fig. 3:**
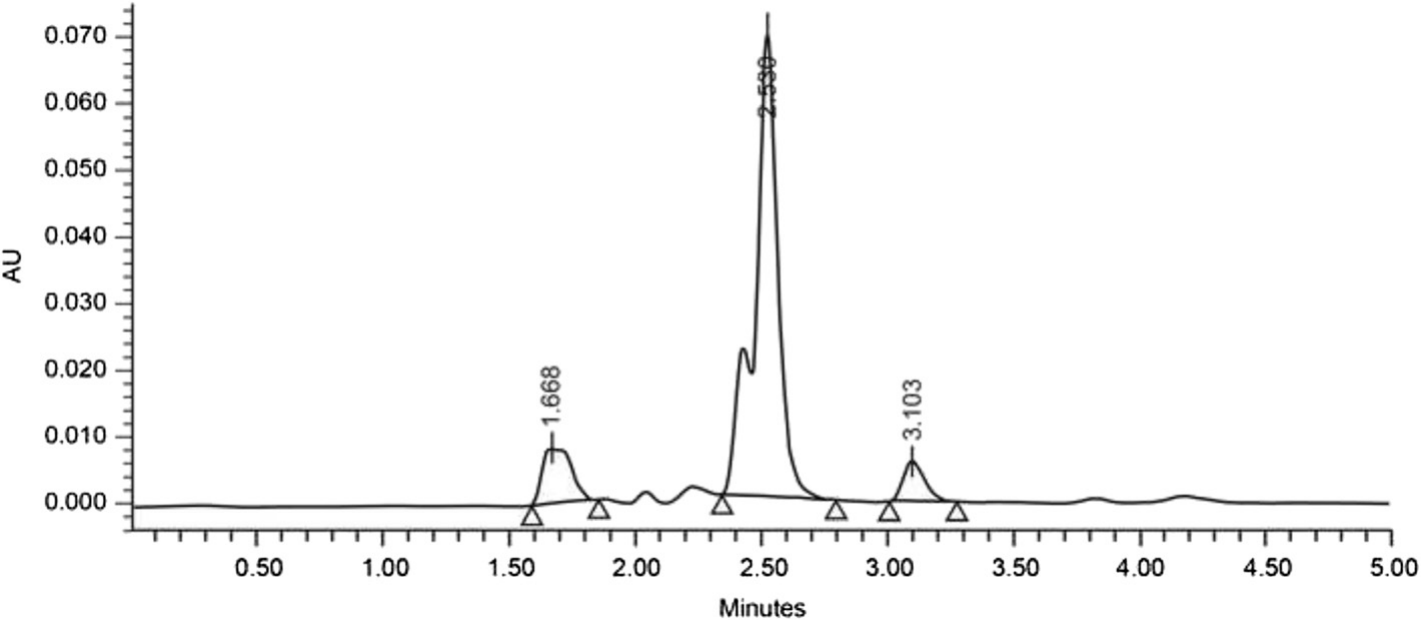
High-pressure liquid chromatogram (measured in UV absorbance units) derived from a plasma sample in which the total vitamin C concentration was 10.7 μmol/L. The ascorbic acid peak eluted 3.1 min after injection onto the column. The two earlier peaks are due to the reagents metaphosphoric acid and EDTA, respectively

The stability of MPA-stabilized samples (for the ascorbic acid measurement) and TCEP-reduced MPA-stabilized samples (for the total vitamin C measurement) were assessed by allowing them to remain in the HPLC autosampler at 4^o^ C for up to 4 h prior to on-column injection (samples are normally injected within one hour). The ascorbic acid signal for un-reduced samples (ascorbic acid) decreased by 3–6 % after 4 h in the autosampler, whereas the signal for TCEP-reduced samples (total vitamin C) remained unchanged for at least 5 h. Figure 3 illustrates a typical UV-HPLC-derived chromatogram.

### Comparison of EC-HPLC and UV-HPLC methods

A control plasma sample was re-analyzed on 10 different days, yielding coefficients of variation for the EC and UV methods of 4.5 % and 3.3 %, respectively (for total vitamin C analysis) and 5.3 % and 4.4 %, respectively (for ascorbic acid analysis). The concentrations (mean ± SD) were as follows: ascorbic acid 104 ± 5.5 μmol/L by EC-HPLC and 107 ± 4.7 μmol/L by UV-HPLC; total vitamin C 106 ± 4.8 μmol/L by EC-HPLC and 110 ± 3.7 μmol/L by UV-HPLC. The lower limit of signal quantization was 1.34 μmol/L with EC-HPLC and < 4.0 μmol/L with UV-HPLC.

**Fig. 4:**
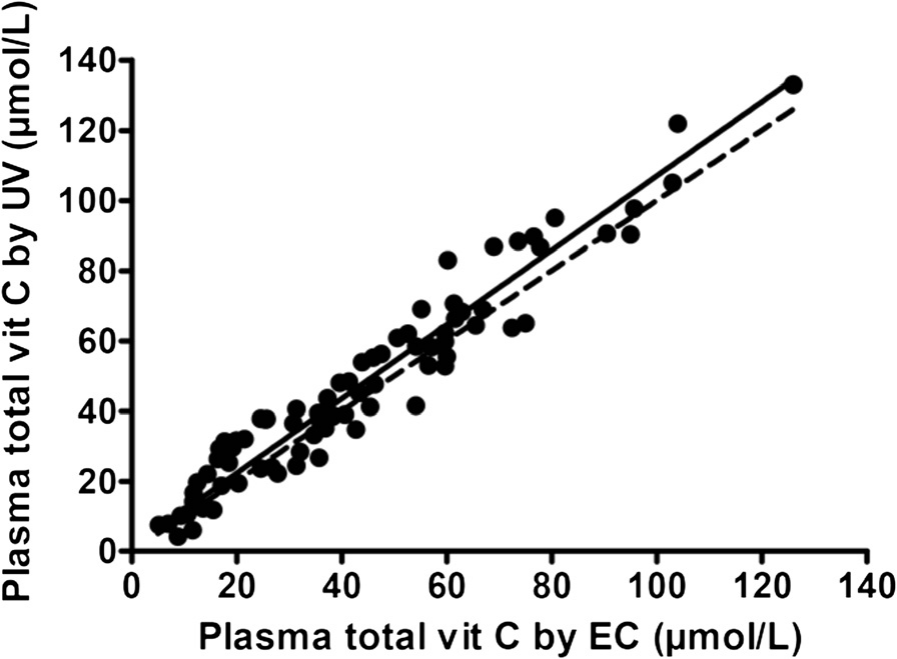
Correlation between the electrochemical (EC) and ultraviolet (UV) detection methods in 80 consecutive clinical samples. The solid line represents the Deming regression line. The dashed line is the line of identity

As shown in Table 1, plasma ascorbic acid and total vitamin C concentrations in 80 consecutive clinical samples determined by both methods ranged from 4–134 μmol/L; the individual values were highly correlated (Spearman r = 0.96, P < 0.0001 both for ascorbic acid and total vitamin C). The Deming regression graph for total vitamin C is shown in Fig. 4. The 95 % confidence interval for the slope of the regression equation encompassed 1 (0.99–1.12) and the corresponding interval for the Y-intercept encompassed zero (-2.15–4.49), indicating that the concentrations reported by the different methods were equal and indistinguishable. There was similarly close agreement for the ascorbic acid analysis.

**Table 1:** Plasma ascorbic acid and total vitamin C concentrations (μmol/L) as measured by EC-HPLC and UV-HPLC in 80 consecutive patient samples

		EC-HPLC	UV-HPLC
Ascorbic acid	Mean ± SD	39.1 ± 24.3	45.2 ± 27.6
	Median (interquartile range)	35.3 (17.1-53.5)	38.1 (25.0-60.0)
	Range	5.80-117	4.10-134
Total vitamin C	Mean ± SD	43.3 ± 26.3	47.0 ± 27.8
	Median (interquartile range)	39.0 (20.0-59.9)	40.0 (26.5-63.3)
	Range	5 - 126	4 - 133

**Fig. 5:**
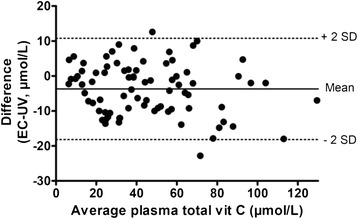
Bland-Altman plot of the data in Fig. 4. Average vitamin C is the sum of the total vitamin C concentrations obtained with the two methods divided by 2

Even a very high correlation does not, in itself, provide sufficient evidence of acceptable agreement between two methods, so a Bland-Altman analysis was carried out to quantify any systematic difference between the two methods [72]. The resulting Bland-Altman plot is shown in Fig. 5. The mean difference (bias) between the EC and UV results was -3.70 μmol/L. Bland-Altman plots are generally interpreted informally and in the context of their clinical significance. For total vitamin C concentrations < 28.4 μmol/L, which represented 24 % of the sample and are the ones of most clinical interest, the bias between the methods was -1.02 μmol/L (not significantly different from zero).

Table 2 compares EC-HPLC as used in our laboratory, the three available UV-HPLC methods [59, 63, 64] for total vitamin C, and the UV-HPLC method described here regarding its use in an ordinary clinical laboratory.

**Table 2:** Comparison of HPLC methods for total plasma vitamin C analysis

	EC^a^	Other UV methods^b^	Present UV method
Equipment			
Availability	Limited	Wide	Wide
Cost	High	Moderate	Moderate
Maintenance	Complex, lengthy	Simple	Simple
Separation column	Dedicated ion-pairing	Reverse phase or ion-pairing	Reverse phase
Number of reagents	14	3-6	4
Reagent preparation time (h)	4	1.5	1
Number of procedures^c^	7	4-6	4
Number of pH adjustments	3	0-2	0
Time prior to sample injection (h)^d^	4-5	2-3	2
Chromatographic run time (min)	8	2-16	6
pH			
Mobile phase	4.8	4.5-5.7	2.7
Low pH throughout procedure	No	No	Yes

^a^As described in the Methods section

^b^As described in [59, 63, 64]

^c^Preparation of reagents and mobile phase, pH adjustments and sample handling

^d^Preparation of mobile phase, system equilibration, reduction reaction and standards and control analysis

## Discussion

UV-HPLC analysis of vitamin C in biological fluids is not novel as a general procedure: it was first reported more than 40 years ago [73] and many variations have subsequently been published [49, 51, 52, 56, 58, 60–67]. However, a variety of methodological complexities and the inability, in all but a few of them [59, 63, 64], to determine the clinically important analyte, total vitamin C, limit their usefulness in the clinical setting.

Total plasma vitamin C is the measurement of choice for two reasons: first, it indicates the total amount of vitamin C in the sample, and second, ascorbic acid can easily oxidize to dehydroascorbic acid in a busy clinical setting due to inadvertent and sometimes unavoidable delays or minor technical shortcomings in sample handling or the analysis procedure. Indeed, there is some evidence that much or most dehydroascorbic acid detected in plasma may be artificially generated during sample handling and processing [49, 74]. Treatment of samples with an appropriate reducing agent helps prevent under-estimation of vitamin C concentrations (and hence over-diagnosis of hypovitaminosis C) due to minor vagaries in sample handling and processing [75]. It is a major advantage of the present method that the sample reduction procedure is extremely simple and trouble-free.

The UV-HPLC method described here for plasma ascorbic acid and total vitamin C analysis demonstrates highly acceptable sensitivity and reproducibility. It is especially notable for easy and rapid setup, analytical column conditioning, fewer reagents and sample manipulations than other comparable methods, and a very simple constant-low-pH sample reduction procedure. The only instruments required are a reverse-phase HPLC column and pre-column and UV detector, standard equipment in hospital laboratories. Although these features are less important in a dedicated research or reference laboratory, they make the method practical for use in an ordinary clinical laboratory.

The mobile phase is extremely simple, stable and isocratic. This type of mobile phase composition was previously reported for analyzing vitamin C in green beans [69], but the procedure had to be modified to account for the much greater sensitivity required for plasma analysis, plasma’s much higher protein concentration (which necessitates a high concentration of the deproteinizing acid) and the need to separate the analyte peak from uric acid. (In fact, the uric acid peak is so well separated that uric acid can be analyzed using this method [62, 66], but it provides no advantage over the automated analysis routinely available in clinical laboratories.) The second major advantage of the present method is the use of the reducing agent TCEP at very low pH [70, 76], which allows extremely simple, trouble-free sample processing and analysis at constant low pH. Other methods for reducing dehydroascorbic acid to ascorbic acid often involve multi-step and potentially error-prone pH adjustments, perhaps contributing to the known problem of inter-laboratory variability in results reported with this analysis [44].

The present method agreed closely with the gold-standard method, EC-HPLC. The Bland-Altman analysis indicated that, on average, plasma total vitamin C concentrations are 3.7 μmol/L higher with the UV method. This small bias did not exist for concentrations below the lower limit of the normal range, 28.4 μmol/L, which are of greatest clinical interest. It is indeterminate whether the UV method slightly over-estimates plasma vitamin C concentrations or the EC method slightly underestimates them. This formal comparison between UV and EC vitamin C detection methods was made on 80 consecutive clinical samples with no samples excluded.

EC-HPLC is widely recommended over UV-HPLC for plasma vitamin C analysis [46, 49, 51, 56], but direct evidence that supports this recommendation is unavailable. The only previous direct comparison of EC-HPLC with UV-HPLC was with regard to ascorbic acid rather than total vitamin C, it was based on only 27 samples from healthy individuals, and it lacked a Bland-Altman analysis [52]. Nevertheless, the close agreement that was reported between EC and UV detection of ascorbic acid, even at low plasma concentrations, is consistent with our observations regarding total plasma vitamin C concentrations in a much larger number of consecutive clinical samples.

A very recent article describes a UV-HPLC method for simultaneously determining ascorbic acid and dehydroascorbic acid in human plasma [68]. The applicability of this method to clinical medicine seems doubtful, because its lower limits of detection for ascorbic acid (11.4 μmol/L) and dehydroascorbic acid (57 μmol/L) are well above the range of clinical interest. Moreover, it indicates a normal plasma dehydroascorbic acid concentration of ~ 50 μmol/L [68], despite the heavy preponderance of evidence that plasma dehydroascorbic acid concentrations seldom exceed 5 μmol/L [77] and under optimum handling and processing conditions are usually close to zero [74].

The UV-HPLC method described here has limitations. We used a standard reverse phase HPLC column (and precolumn) which demonstrated excellent performance for more than 1 year during which a large numbers of samples were injected onto it. In situations in which large numbers of samples are analyzed, polymeric or polar embedded reverse phase columns could be considered, for they are more resistant to long-term degradation caused by a completely aqueous, highly acidic mobile phase [56]. Another potential problem is a greater chance of interference by exogenous molecules than EC-HPLC [51, 56]. This problem has not been reported during 30 years of UV-based plasma vitamin C analysis, but hospitalized patients receive many different drugs. Plasma vitamin C concentrations could be overestimated if a small exogenous molecule absorbed UV light at 245 nm and eluted from the HPLC column at precisely the same time as ascorbic acid.

## Conclusion

In-hospital hypovitaminosis C is highly prevalent, potentially serious, and easily preventable, but most physicians remain unaware of it. The unavailability of simple and reliable plasma vitamin C determinations in hospitalized patients creates a barrier to medical awareness and investigation of this potentially important phenomenon. We describe a method that fulfills these requirements by making technical improvements to existing UV-HPLC procedures and demonstrating that the resulting method performs equivalently to the gold-standard method, EC-HPLC.
