# Erratum to: ‘A simple method for plasma total vitamin C analysis suitable for routine clinical laboratory use’

**DOI:** 10.1186/s12937-016-0171-z

**Published:** 2016-05-05

**Authors:** Line Robitaille, L. John Hoffer

**Affiliations:** Lady Davis Institute for Medical Research and Department of Medicine, McGill University and Jewish General Hospital, 3755 Cote Sainte Catherine, Montreal, QC H3T 1E2 Canada

Unfortunately, the original version of this article [1] contained errors. Two editing changes were made incorrectly which led to the resulting text containing confusing grammatical errors.

On page three, the line that contains the following:

Zero-degree thawed omit plasma samples were divided into two aliquots of 30 μL in 0.6 mL plastic Eppendorf tubes.

Should read as:

Zero-degree thawed samples were divided into two aliquots of 30 μL in 0.6 mL plastic Eppendorf tubes.

On page six, the line that contains the following:

[…] it provides no advantage over the automated analysis omit that’s routinely available in clinical laboratories.)

Should read as follows:

[…] it provides no advantage over the automated analysis routinely available in clinical laboratories.)
